# The Ecological Dynamics of Fecal Contamination and *Salmonella* Typhi and *Salmonella* Paratyphi A in Municipal Kathmandu Drinking Water

**DOI:** 10.1371/journal.pntd.0004346

**Published:** 2016-01-06

**Authors:** Abhilasha Karkey, Thibaut Jombart, Alan W. Walker, Corinne N. Thompson, Andres Torres, Sabina Dongol, Nga Tran Vu Thieu, Duy Pham Thanh, Dung Tran Thi Ngoc, Phat Voong Vinh, Andrew C. Singer, Julian Parkhill, Guy Thwaites, Buddha Basnyat, Neil Ferguson, Stephen Baker

**Affiliations:** 1 Oxford University Clinical Research Unit, Patan Academy of Health Sciences, Kathmandu, Nepal; 2 MRC Centre for Outbreak Analysis and Modelling, Department of Infectious Disease Epidemiology, School of Public Health, Imperial College London, London, United Kingdom; 3 The Wellcome Trust Sanger Institute, Hinxton, Cambridgeshire, United Kingdom; 4 Microbiology Group, The Rowett Institute of Nutrition and Health, University of Aberdeen, Aberdeen, United Kingdom; 5 The Hospital for Tropical Diseases, Wellcome Trust Major Overseas Programme, Oxford University Clinical Research Unit, Ho Chi Minh City, Vietnam; 6 Centre for Tropical Medicine, Oxford University, Oxford, United Kingdom; 7 Grupo de Investigación Ciencia e Ingeniería del Agua y el Ambiente, Facultad de Ingeniería, Pontificia Universidad Javeriana, Bogotá, Colombia; 8 NERC Centre for Ecology and Hydrology, Wallingford, Oxfordshire, United Kingdom; 9 The London School of Hygiene and Tropical Medicine, London, United Kingdom; University of Otago, NEW ZEALAND

## Abstract

One of the UN sustainable development goals is to achieve universal access to safe and affordable drinking water by 2030. It is locations like Kathmandu, Nepal, a densely populated city in South Asia with endemic typhoid fever, where this goal is most pertinent. Aiming to understand the public health implications of water quality in Kathmandu we subjected weekly water samples from 10 sources for one year to a range of chemical and bacteriological analyses. We additionally aimed to detect the etiological agents of typhoid fever and longitudinally assess microbial diversity by 16S rRNA gene surveying. We found that the majority of water sources exhibited chemical and bacterial contamination exceeding WHO guidelines. Further analysis of the chemical and bacterial data indicated site-specific pollution, symptomatic of highly localized fecal contamination. Rainfall was found to be a key driver of this fecal contamination, correlating with nitrates and evidence of *S*. Typhi and *S*. Paratyphi A, for which DNA was detectable in 333 (77%) and 303 (70%) of 432 water samples, respectively. 16S rRNA gene surveying outlined a spectrum of fecal bacteria in the contaminated water, forming complex communities again displaying location-specific temporal signatures. Our data signify that the municipal water in Kathmandu is a predominant vehicle for the transmission of *S*. Typhi and *S*. Paratyphi A. This study represents the first extensive spatiotemporal investigation of water pollution in an endemic typhoid fever setting and implicates highly localized human waste as the major contributor to poor water quality in the Kathmandu Valley.

## Author Summary

Aiming to understand the ecology of municipal drinking water and measure the potential exposure to pathogens that cause typhoid fever (*Salmonella* Typhi and *Salmonell*a Paratyphi A) in Kathmandu, Nepal, we collected water samples from 10 water sources weekly for one year and subjected them to comprehensive chemical, bacteriological and molecular analyses. We found that Kathmandu drinking water exhibits longitudinal fecal contamination in excess of WHO guidelines. The chemical composition of water indicated site-specific pollution profiles, which were likely driven by localized contamination with human fecal material. We additionally found that *Salmonella* Typhi and *Salmonell*a Paratyphi A could be detected throughout the year in every water sampling location, but specifically peaked after the monsoons. A microbiota analysis (a method for studying bacterial diversity in biological samples) revealed the water to be contaminated by complex populations of fecal bacteria, which again exhibited a unique profile by both location and time. This study shows that *Salmonella* Typhi and *Salmonell*a Paratyphi A can be longitudinally detected in drinking water in Kathmandu and represents the first major investigation of the spatiotemporal dynamics of drinking water pollution in an endemic typhoid setting.

## Introduction

Enteric (typhoid) fever is a severe systemic infection and a common cause of community acquired febrile disease in many low-income countries in Asia and Africa [1]. The infection is triggered by the ingestion of the bacteria *Salmonella* Typhi (*S*. Typhi) and *Salmonella* Paratyphi A (*S*. Paratyphi A). Both *S*. Typhi and *S*. Paratyphi A are human restricted pathogens (they have no known animal reservoir) and is it acknowledged that they are transmitted through contaminated food and water or via contact with fecal matter from acute or chronically infected individuals [1]. However, the predominant route of infection has never been rigorously investigated in an endemic setting outside a conventional case/control study design [2,3].

**Fig 1:**
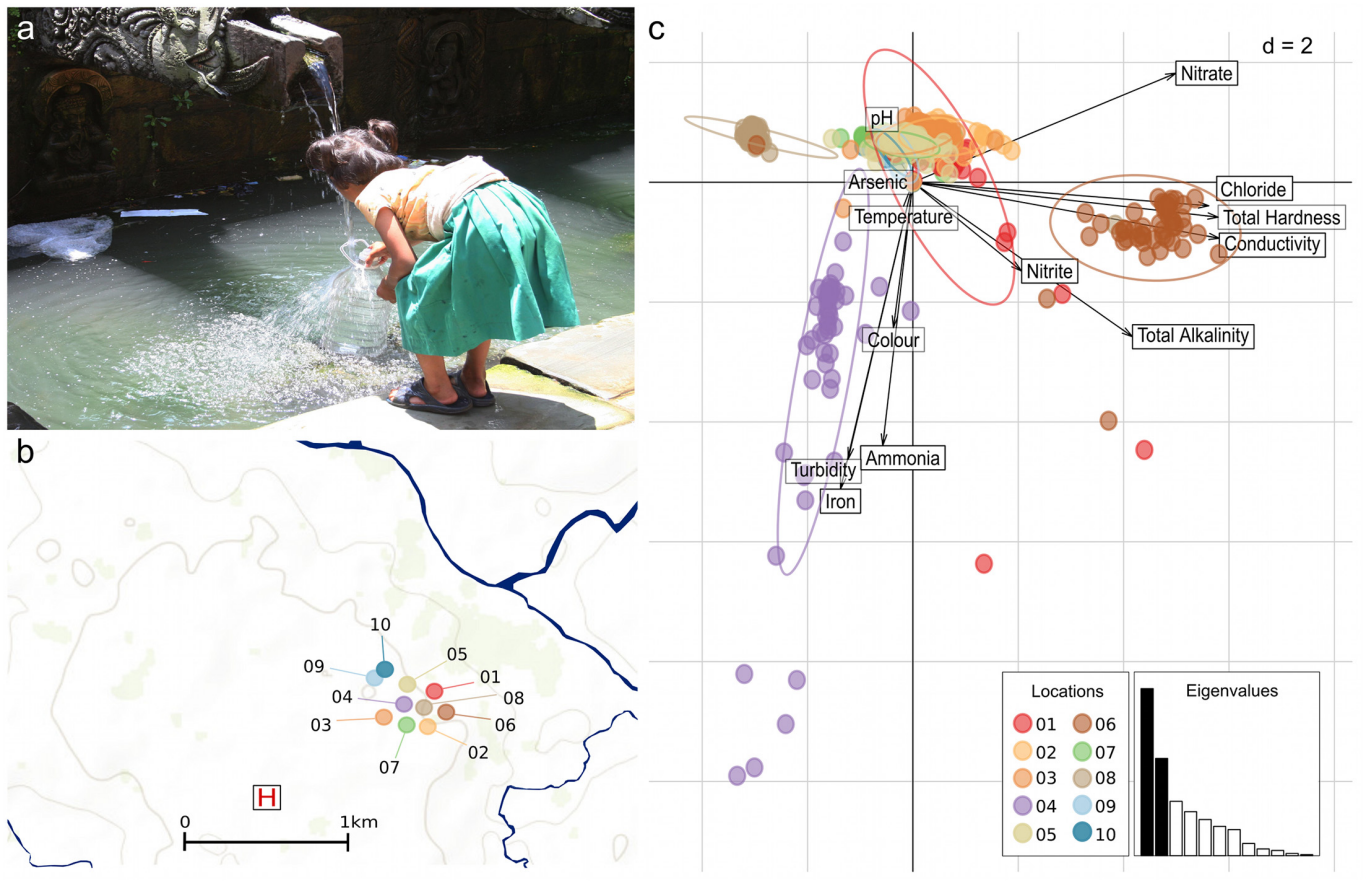
The physical and chemical properties of water from ten sampling locations in Kathmandu, Nepal. a) Photograph of child collecting water from a traditional stone waterspout (dhunge dhara) in Kathmandu. b) A map showing the location of the ten sampling locations in Kathmandu. The locations are color-coded corresponding to other figures and the site of Patan hospital (the healthcare facility for those with typhoid fever) is highlighted by the H symbol. (See Table 1 and Baker *et al*. [6] for more details regarding the sampling locations and the mapped area). c) Scaled principal components analysis of the physical and chemical properties of water samples from the ten sampled locations, showing the first two principal components (PC1-2). Individual water sample are identified by dots colored according to their sampling location (see Fig 1a). Inertia ellipses indicate the overall distribution of each water source. Labeled arrows indicate the relative contributions of the variables to the principal components, with longer arrows reflecting larger contributions. The screeplot of eigenvalues (inset) indicates the amount of variation contained in the different principal components, with PC1 and PC2 indicated in black.

Typhoid fever is a common infection in Kathmandu (the capital city of Nepal) and our previously generated serological data implies that the local population has longitudinal exposure to both of these endemic pathogens [3,4]. Further studies, generated through investigating the spatiotemporal dynamics of typhoid fever in Kathmandu predicted that both *S*. Typhi and *S*. Paratyphi A are more likely to be transmitted through contaminated water than via human-to-human transmission in this setting [5,6]. Significantly, we found that typhoid fever cases cluster in areas with a high density of urban water sources, which are gravity driven and, therefore, rationally located at lower elevations. The various urban water sources in Kathmandu are most commonly in the form of sunken wells (as found in many urban and rural settings in lower-income countries), piped supplies into large communal holding tanks or the more traditional stone waterspouts (hitis/dhunge dharas) [7]. The iconic stone spouts are common across the Nepalese capital and the water flow into these sacred locations is gravity-dependent (Fig 1a), replenished by rainfall and snowmelt from the surrounding Himalayan Mountains. Natural soft-rock aquifers act as reservoirs for ground water and ultimately the untreated water enters the stone spouts from the aquifers through a series of ancient porous underground channels. Our previous analysis specifically identified locations around these stone spouts are hotspots for *S*. Typhi and *S*. Paratyphi A infections [5,6].

Hypothesizing that the local water, particularly the water accessed via the stone spouts, is a substantial public health risk for typhoid fever and other enteric infections in Kathmandu, we aimed to longitudinally assess bacterial contamination, the chemical composition and the ecological dynamics of enteric bacteria in the water supply in this location. To address this hypothesis, and focusing on water sources accessed by the local population, weekly water samples were collected over a one-year sampling period from ten locations and subjected to various physical, chemical, microbiological and molecular analyses.

## Methods

### Water sampling locations

The water sources for this study were the ten most commonly used water sources (identified by questionnaire) lying within a previously identified typhoid fever hotspot in Lalitpur, Kathmandu [6]. The selected locations were GPS located using an eTrex legend (Garmin) and consisted of five stone spouts, three sunken wells and two piped supplies. The location of these water sources are shown in Fig 1b and described in detail in Table 1. Daily rainfall data from Kathmandu Airport was provided by the Nepalese Department of Hydrology and Meteorology (http://www.dhm.gov.np/) and aggregated into weeks for the purposes of the analysis presented here.

**Table 1:** Water sampling locations in this study.

Location	Source type	Latitude	Longitude	Depth to water (m)	Source	Treatment	Additional protection	Samples collected/weeks	16S rRNA gene analysis
1	Stone spout	N 27°40.482	E 85°19.693	Ground level	Shallow aquifer	Untreated	None	40/53	No
2	Stone spout	N 27°40.461	E 85°19.693	Ground level	Shallow aquifer	Untreated	None	38/53	Yes
3	Stone spout	N 27°40.426	E 85°19.51	Ground level	Shallow aquifer	Untreated	None	51/53	No
4	Sunken well	N 27°40.454	E 85°19.665	8.4	Shallow aquifer	Untreated	Mesh covered	51/53	No
5	Sunken well	N 27°40.503	E 85°19.660	7.1	Shallow aquifer	Untreated	Mesh covered	50/53	Yes
6	Sunken well	N 27°40.545	E 85°19.719	8.1	Shallow aquifer	Untreated	Mesh covered	50/53	No
7	Piped supply	N 27°40.422	E 85°19.645	Ground level	Delivery truck	Irregular chlorination	Locked container	51/53	No
8	Piped supply	N 27°40.457	E 85°19.698	Ground level	Delivery truck	Irregular chlorination	Locked container	50/53	No
9	Stone spout	N 27°40.607	E 085°19.540	Ground level	Shallow aquifer	Untreated	None	21/53	No
10	Stone spout	N 27°40.607	E 085°19.540	Ground level	Shallow aquifer	Untreated	None	30/53	No

### Collection of water samples for analysis

Water was collected (when permitted by water flow), from all of the 10 locations once per week over one year from May 2009 to April 2010. From each of the sources mid-flow water samples were collected in two sterile bottles in volumes of 1 L and 500 ml. From the stone spouts and the piped supply, the stopper was aseptically removed and free flowing water was allowed to flow directly into the sterile bottle. For wells, a sterilized steel bucket (bleached and washed with autoclaved water prior to use) was lowered into the well until it was partially submerged, the bucket was then removed and the water was poured into the bottles. All the bottles were labeled with the source code, date and time of collection of the samples. After recording the water temperature the bottles were transported to the laboratory at ambient temperature and were processed within one hour for physical, chemical and microbiological analysis.

### Physical and chemical analysis of water samples

The Kathmandu Water Engineering Laboratory (http://www.sodhpuch.com/water-engineering-training-centre-p.) performed all chemical and physical analyses following their standard operating procedures for international water quality. The measured variables were pH (Hanna pH meter, calibrated with pH4, pH7 and pH9 buffers), temperature (Hanna digital thermometer with probe), conductivity (Hanna conductivity meter), color (Perkin Elmer’s LAMDA 650 UV spectrophotometer at 270 nm) turbidity (NEPHELOstar Plus nephlometer), hardness (EDTA titration), total alkalinity as CaCO_3_ (methyl orange), chloride (argentometric titration), ammonia (nesslerisation), total nitrate (Perkin Elmer’s LAMBDA 650 UV spectrophotometer at 275 nm), total nitrite (Perkin Elmer’s LAMDA 650 UV spectrophotometer at 275 nm), and trace elements and heavy metals (Atomic Absorption Spectrophotometric (AAS) method). All variables were recorded on the day of sampling and compared to WHO guidelines for water quality [8].

### Most Probable Number (MPN) method for measuring thermotolerant coliforms

A modified most probable number (MPN) method was used to assess the microbiological quality of the water, specifically coliform contamination [9,10]. Briefly, five ten-fold serial dilutions were made from each water sample by inoculating 1 ml of undiluted water sample into 9 ml of MacConkey broth (Oxoid, UK). This was continued until a dilution of 1 x 10^−5^. A total of 30 tubes (five tubes for each dilution) were prepared for each sample. The inoculated broths were incubated at 44°C for 48 hours for the culture of thermotolerant coliforms. After incubation, each tube was examined and those that were positive (production of acid and gas) were counted. The number of positive and negative tubes in each of these three sets was noted in order and these data were used to estimate the coliform content using a five-tube MPN table.

### Direct and enrichment plating for enteric bacteria in water

To detect the presence of enteric bacteria with pathogenic potential (e.g. *Salmonellae*, *Shigellae*, *Vibrionaceae*, and *E*. *coli*) 10, 20, 50, 100 and 500 μl of undiluted water was directly plated onto Xylose lysine deoxycholate (XLD) and MacConkey agar plates. The plates were incubated at 37°C overnight and then observed for growth. To increase the likelihood of culturing *Salmonellae* and *Shigellae*, 100 ml of undiluted water was filtered through a membrane filter with a pore size 0.45 μm (Whatman, GE Life Sciences, PA, USA) using a sterile syringe. The filter paper was removed using sterile forceps and placed in 90 ml of typtic soya broth (Oxoid, UK). The soya broth bottles were agitated using a vortex to displace the organisms on the membrane and incubated for 18 hours at 37°C. After overnight incubation 1 ml of the pre-enrichment culture was transferred to 10 ml of selenite broth. Further, 1 ml of the pre-enrichment culture was transferred to 10 ml of Rappaport-Vassiliadis Broth (RVB). The incubated overnight broth was then plated onto XLD and MacConkey agar plates. The plates were incubated overnight at 37°C and then observed for growth. For the detection of *Vibrionaceae*, 1 ml of the undiluted water sample was diluted in 9 ml of alkaline peptone water. The suspension was then incubated overnight at 37°C and then plated on to MacConkey, XLD and thiosulphate-citrate-bile salts sucrose (TCBS) agar.

### Bacterial identification

The colony morphologies including the form, size, surface appearance, texture, color, elevation and margin of all individual colonies were recorded from MacConkey and XLD plates. Of special interest were colonies that were circular, with an entire margin and slightly raised elevation that were non-lactose fermenting on both plates, with or without the production of hydrogen sulphide on the XLD plate. Individual colonies with the aforementioned characteristics were isolated and plated on nutrient agar and incubated at 37°C for 24 hours. Isolated colonies obtained on the nutrient agar plates were then subject to API20E testing to identify *Enterobacteriaceae* and other non-fastidious Gram-negative rods.

### Total DNA extraction from water samples

Total DNA from all water samples was extracted using the Metagenomic DNA Isolation Kit for Water (Epicentre Biotechnologies, WI, USA). Water samples were centrifuged at 1,000 rpm (Hettich Zentrifugen, EBA 21, Germany) for 5 minutes to remove large debris and then decanted into sterile containers. After centrifugation, 100 ml of the centrifuged water was filtered through a pre-sterilized filter with a pore size of 0.45 μm (Whatman, GE Life Sciences, PA, USA). Using sterile forceps and scissors the membrane was removed from the filter apparatus and cut into four pieces. The cut filters were then placed in a 50 ml sterile conical tube with the upper surface of the filter facing inwards. One milliliter of filter wash buffer containing 0.2% Tween-20 was added to the filter pieces in the tubes to remove organisms on the filter surface. The tube was agitated at high speed for approximately 2 minutes with intermittent breaks. The cell suspension was transferred to a clean micro-centrifuge tube and centrifuged at 14,000 X g (Thermo Fischer Scientific, IEC Micro CL17, Germany) for 2 minutes to pellet the cells. The supernatant was discarded. The cell pellet was re-suspended in 300 μl of TE buffer, and 2 μl of ready-lyse lysozyme solution and 1μl of RNAse A were added and mixed thoroughly. The tube was incubated at 37°C for 30 minutes and then 300 μl of 2 X meta-lysis solutions and 1 μl of Proteinase K were added to the tube and thoroughly mixed by vortexing. To ensure that all the solution was at the bottom of the tube, the tube was pulse centrifuged. The tubes were then incubated at 65°C for 15 minutes. The solution was cooled to ambient temperature and placed on ice for 5 minutes. 350 μl of MPC protein precipitation reagent was added to the tube and mixed thoroughly by vortexing vigorously for 10 seconds. The debris was pelleted by centrifugation for 10 minutes at 14,000 X g (Thermo Fischer Scientific, IEC Micro CL17, Germany) at 4°C. The supernatant was transferred to a clean micro-centrifuge tube and the pellet was discarded. To the supernatant, 570 μl of isopropanol was added and mixed by inverting the tube multiple times. The DNA was pelleted by centrifugation for 10 minutes at 14,000 X g (Thermo Fischer Scientific, IEC Micro CL17, Germany) at 4°C. The isopropanol was removed and the sample was briefly pulse centrifuged and any residual liquid was removed without disturbing the pellet. To the pellet 500 μl of 70% ethanol was added without disturbing the pellet. The tube was then centrifuged for 10 minutes at 14000 X g (Thermo Fischer Scientific, IEC Micro CL17, Germany) at 4°C. Ethanol was removed without dislodging the DNA pellet and the sample was briefly pulse centrifuged and any residual fluid was removed without disturbing the pellet. The pellet was then air dried for 8 minutes at ambient temperature before being resuspended in 100 μl of nucleic acid free sterile water (Epicentre Biotechnologies, WI, USA).

### Real-time polymerase chain reaction to detect *S*. Typhi and *S*. Paratyphi A in water samples

Quantitative Real-time PCR was performed on all extracted DNA to detect DNA sequences specific for *S*. Typhi and *S*. Paratyphi A as previously described [11]. Primer and probe sequences were as follows; *S*. Typhi; ST-Frt 5' CGCGAAGTCAGAGTCGACATAG 3', ST-Rrt 5' AAGACCTCAACGCCGATCAC 3', ST- Probe 5' FAM-CATTTGTTCTGGAGCAGGCTGACGG-TAMRA 3'; *S*. Paratyphi A; Pa-Frt 5'ACGATGATGACTGATTTATCGAAC 3', Pa-Rrt 5' TGAAAAGATATCTCTCAGAGCTGG 3', Pa-Probe 5' Cy5-CCCATACAATTTCATTCTTATTGAGAATGCGC-BHQ5 3'. Briefly, 5 μl of environmental DNA extractions (as above from 100 ml of water and resuspended in 100 μl of nucleic acid free sterile water) was used as the template for each experiment, i.e. 5 μl equated to 5 ml of water sample. Quantification was performed using standard curves where plasmid DNA carrying the target sequences were diluted in 10-fold serial dilutions ranging from 10^0^ to 10^5^ plasmid copies per μl; standard curves for assessing *S*. Typhi and *S*. Paratyphi A copy number were constructed by plotting the *Ct* value against the plasmid DNA copy number.

### 16S rRNA gene amplification and sequencing

For the 16S rRNA gene surveying, variable regions 3 to 5 (V3–V5) of the 16S rRNA gene were PCR amplified from the water DNA extractions. The primers used were as described previously [12], see S1 Table for the full barcode and primer sequences (30 nucleotides for 454 adaptor, 12 nucleotides for unique recognition (tag) and 18 nucleotides to amplify the specific V3–V5 region) used for each sample in the present study. The conditions of PCR were as follows: 1U of AccuPrime Taq DNA Polymerase High Fidelity (Invitrogen, Carlsbad, CA USA), 200 mM of forward and reverse primer, 2 μl of template environmental DNA in a 20 μl reaction. The reaction was cycled for 1 x 94°C for 2 minutes, and then 20x (94°C for 30 seconds, 53°C for 30 seconds and 68°C for 2 minutes). Each sample was PCR amplified on four occasions, the resulting amplicons were pooled and then ethanol precipitated before resuspension in 20 μl of TE. The 16S rRNA gene amplicons were shipped to The Wellcome Trust Sanger Institute and pooled together into an equimolar mastermix, as measured by a Qubit fluorometer (Invitrogen, Carlsbad, CA, USA), prior to sequencing on a GS FLX Titanium 454 machine (Roche Diagnostics, Oakland, CA USA) using the Lib-L kit. The resulting sequence data is available at the European Nucleotide Archive under Study Accession Number ERP004371/Sample Accession number ERS373486. Sequence data was processed using the mothur software package (http://www.mothur.org/), following a previously described protocol [12]. This removed poor quality reads, and generated taxonomic classifications for each Operational Taxonomic Unit (OTU). Following these filtering steps 326,155 sequences remained (range of 1 to 7,396 sequences per sample).

### Statistical analysis of bacterial contamination data

We first tested for geographic differences in bacterial assays, using the non-parametric MANOVA implemented in the package *ade*4 [13] for the R software suite [14]. 9,999 random permutations of the data were used to assess statistical significance of Pillai’s statistic [15] and compute the associated *p*-value. After ruling out the presence of geographic differences between samples, data were aggregated across locations by computing average weekly profiles, from which temporal trends were more straightforward to investigate. As bacterial assays may each capture different aspects of water contamination, these data were subjected to a centered Principal Component Analysis (PCA) [13], which we used to derive a latent variable (the first principal component, PC1) as correlated as possible to all the different assays [16] and therefore reflecting the extent of bacterial contamination. PCA is ideally suited to derive synthetic variables, which capture the essential trends of variation in quantitative or binary data, and is thus readily applicable to water quality data including physical chemical properties, bacterial assays and meta-genomic variation.

The temporal trends in PC1 were visualized using ggplot2 [17] and modeled using a cubic spline of sample collection dates. Five breakpoints were used in the model as they gave the best visual fit, and no other number of breakpoints led to significantly better models. This model was compared to a model where PC1 was constant in time using a classical ANOVA comparing the residual variances of the two models.

### Statistical analysis of chemical pollution data

**Fig 2:**
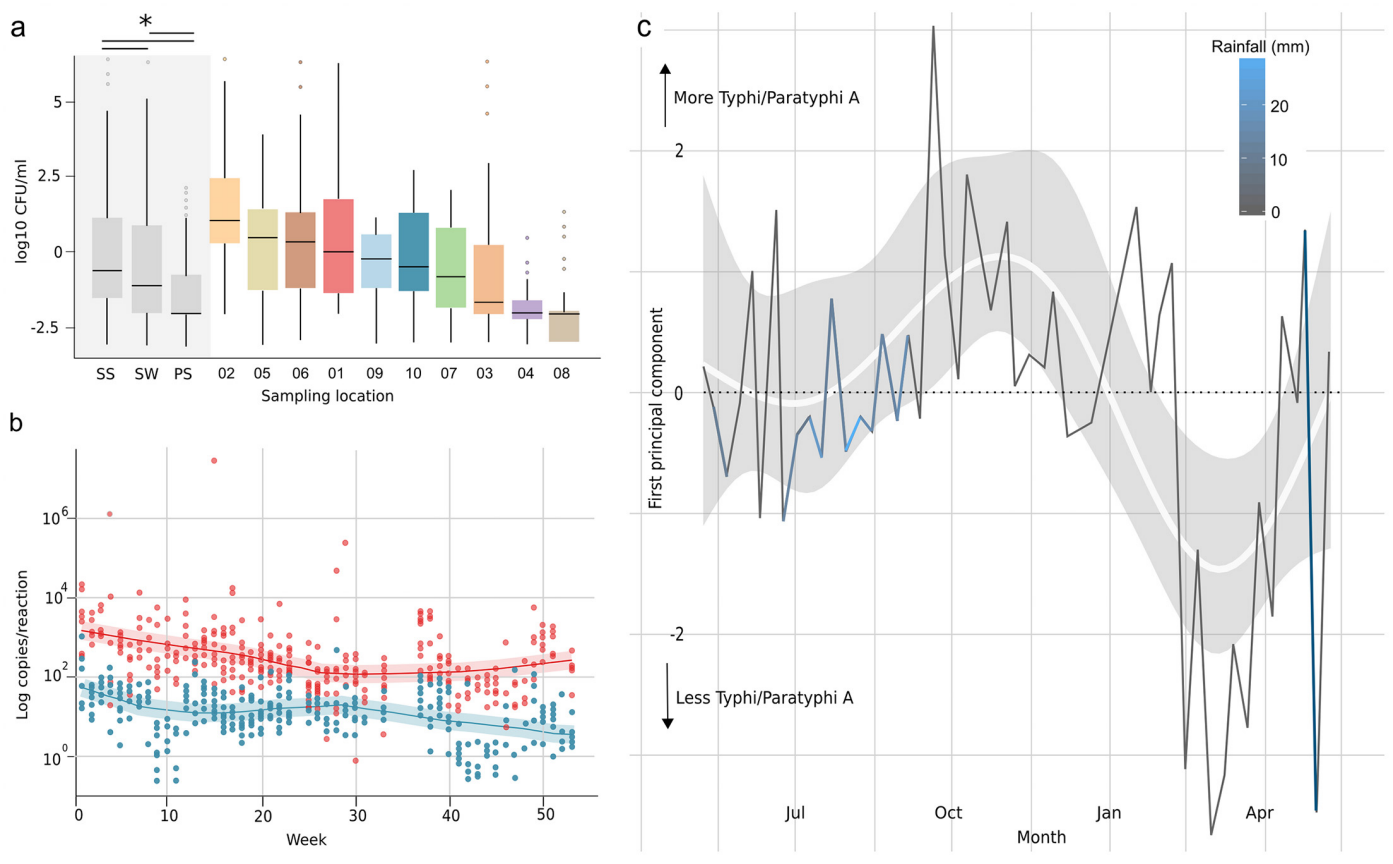
The quantification of coliforms and DNA from *Salmonella* serovars Typhi and Paratyphi A in water samples from ten locations in Kathmandu, Nepal. a) Boxplots of the MPN counts (log10 CFU/ml) aggregated by water sources type (SS, stone spout; SW, sunken well; PS, piped supply) and within each individual location (numbered on x-axis). Boxes and horizontal lines represent the interquartile ranges and medians, respectively and whiskers represent 90% of data range. The median MPN measurements in water from stone spouts; sunken wells and piped supplies were significantly different; as determined by Kruskal Wallis test (*p<*0.001; shown by the asterisk). b) Scatterplots showing the number of copies of *S*. Typhi (red circles) and *S*. Paratyphi A (blue circles) gene targets calculated to be in each water sample (by realtime PCR amplification and quantification using a standard curve) against the week of sampling. The red and blue shaded lines show the best fit through time of gene copy quantification trends for *S*. Typhi and *S*. Paratyphi A, respectively. c) Principal components analysis of the weekly presence/absence profiles of *S*. Typhi and *S*. Paratyphi A gene targets in water samples, shaded by weekly rainfall (see key). Time is represented on the x-axis. The first principal component, representing fluctuations in *S*. Typhi and *S*. Paratyphi A DNA relative abundance, is plotted on the y-axis.

As for bacterial assays, the various chemical properties measured captured potentially different aspects of water pollution. PCA was used to identify the main trends of variation amongst water samples [18,19]. Because measurements were made using different units and had inherently different scales of variation, a centered and scaled PCA was used [20]. Missing data were replaced by the average of the corresponding variables, as is customary in PCA [20]. Results of this first PCA were driven by an outlier, which turned out to be a sample of exceptionally poor water quality. In such cases, because PCA finds linear combinations of variables with maximum variance, the presence of an outlier may conceal other interesting structures [20]. As a consequence, this sample was removed in a second PCA, the results of which are shown in Fig 2. Differences in water chemistry of the water sources were tested using the same MANOVA procedure used in bacterial assays analysis.

### Spatiotemporal dynamics of bacterial composition from 16S rRNA gene data

**Fig 3:**
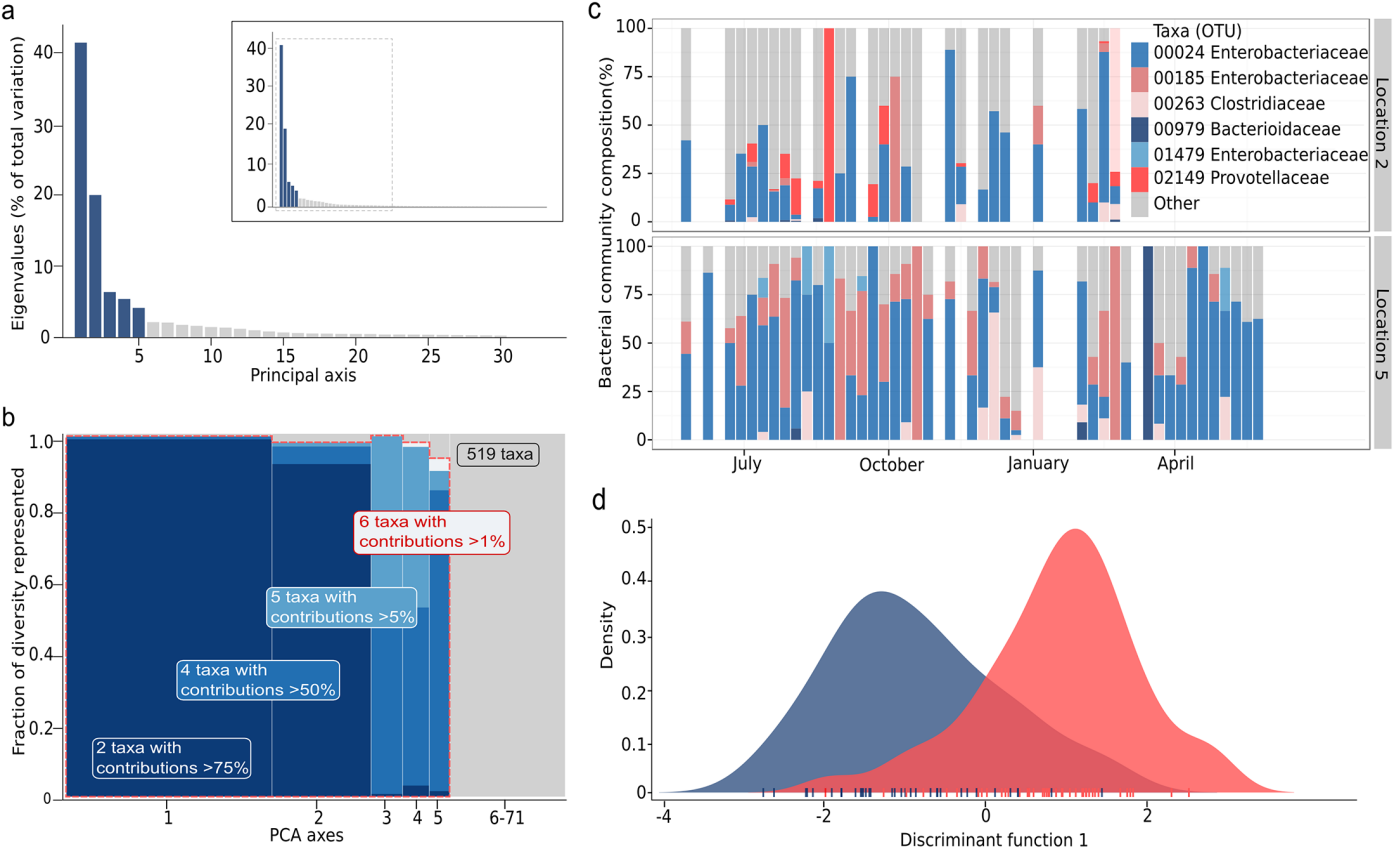
16S rRNA gene surveying of gastrointestinal bacterial taxa found in stone spout and well water samples in Kathmandu, Nepal. a) Screeplot showing the eigenvalues of the PCA of 16S rRNA gene data, with retained axes in blue corresponding to 80% of the variation in 519 identified OTUs from fecal bacterial families (*Bacteroidaceae*, *Clostridiaceae*, *Enterobacteriaceae*, *Erysipelotrichaceae*, *Lachnospiraceae*, *Lactobacillaceae*, *Prevotellaceae*, *Ruminococcaceae* and *Veillonellaceae*) identified in 93 water samples from location 2 (stone spout) and location 5 (sunken well). The main graph only represents the first 30 eigenvalues (full graph provided in inset). b) Diversity represented by the OTUs with large contributions to the retained PCA axes. PCA axes are represented on the x-axis, with a width proportional to the corresponding diversity (eigenvalue). The y-axis represents the amount of diversity retained by retaining only OTUs with contributions of at least 1% (6 taxa), 5% (5 taxa), 50% (4 taxa) or 75% (2 taxa), indicated by differing shades of blue. The total surface of a given color is proportional to the fraction of the total diversity represented by this set of taxa. The red dashed line identifies the set of 6 retained taxa plotted in Fig 3c, representing 76% of the total variation in the entire data. c) OTU composition of the water samples, showing the relative frequencies of the six most structuring fecal bacterial taxa identified in Fig 3b (*Enterobacteriaceae* (OTUs 00024, 00185 and 01479), *Bacteroidaceae* (OTU00979), *Clostridiaceae* (OTU00263), and *Prevotellaceae* (OTU 2149)). Their relative abundance (y-axis) is represented through time in location 2 (stone spout) and location 5 (sunken well), the two locations with the greatest estimated coliform contamination by MPN. Empty bars correspond to missing or failed samples. d) A Discriminant Analysis of Principal Components (DAPC) of the 16S rRNA gene identifying combinations of the 519 gastrointestinal OTUs differing the most between the stone spout (blue) and the sunken well (red). The OTUs exhibiting the greatest variation between these locations were OTU00263; *Clostridium*, OTU00185; *Enterobacteriaceae*, OTU00979; *Bacteroides* and OTU00024, *Enterobacteriaceae*).

16S rRNA gene survey data consisting of 93 samples and 11,212 OTUs were first transformed into compositional data [21], so that each sample was transformed into a composition of OTUs frequencies summing to 1. This transformation ensures that further analysis will only reflect differences in taxa composition, and not in absolute quantities of sequenced DNA. The Gini-Simpson index was computed for every sample as $1-\sum_{i}p_{i}^{2}$ where *p*_*i*_ is the relative frequency of OTU *i* in the sample. A centered PCA was used to analyze the metagenomic profiles, retaining the first five principal axes as they expressed most of the structured variation in the data (Fig 3a). The proportion of the total variation represented by the *j*^th^ principal axis was computed as *λ*_*j*_/∑_*j*_ *λ*_*j*_ (Fig 3b). The contribution of an OTU *i* to a principal axis *U* defined by a vector of loadings [*u*_1_,*u*_2_,…,*u*_11,212_] was computed as *u*_*i*_^2^, which is justified by the fact that *U* has a norm of 1 (thus ∨*U*∨^2^ = ∑_*i*_*u*_*i*_^2^ = 1). The OTUs contributing most to the retained principal axes were defined as OTUs with contributions greater than a given threshold (Fig 3c). Different sets of OTUs were defined using thresholds of 1%, 2%, 5%, 10%, 20%, 30%, 40%, and 50%, which resulted in retention of between 4 and 14 OTUs. A threshold of 10% was retained as it allowed for conserving essentially all of the variation of the 7 principal axes with only 10 OTUs, which still represented 80% of the total variation in the data.

Discriminant Analysis of Principal Components (DAPC, [22,23]) was applied to the 16S rRNA gene data to identify combinations of OTUs that differed most between stone spouts and well. While originally developed for genetic markers data, this method has since been applied to various other types of data, including 16S rRNA (e.g. [24,25]). Cross-validation was used to assess the optimal number of PCA axes to retain in the preliminary dimension-reduction step, using 100 independent replicates for each number of retained PCA axes and 70% of the samples as training set. The same analyses were repeated to investigate possible differences across the three locations.

## Results and Discussion

### The chemical and physical properties of Kathmandu drinking water

A summary of the chemical and bacterial data generated for each of the 10 sampling locations (432 water samples) is presented in Table 2 (total data available in S1 Dataset). We firstly assessed the physical qualities and chemical composition of the water samples and compared these data to WHO guidelines (Table 2) [8]. The chemical analyses of the water samples signified that several sources had concentrations of iron and ammonia in excess of WHO guidelines, but notably, only nitrate levels and turbidity consistently exceeded WHO recommendations in all locations. These finding, with respect to nitrate were broadly consistent with previous single time point observations in this location [26,27]. Further investigation identified significant differences in the chemical profiles of the water from the various locations (non-parametric MANOVA: *Λ*_*pillai*_ = 0.250,*p* = 1×10^−4^ with 9,999 permutations). These disparities in chemical compositions could be summarized using a PCA, with water from two of the sunken wells (locations 4 and 6 (Table 1 and Fig 1b)) forming distinct clusters with independent chemical signatures (Fig 1c). These profiles were characterized by consistently high concentrations of iron and ammonia and greater turbidity at location 4 and greater conductivity, hardness, chlorides and nitrates at location 6 (Fig 1c). Distinctively, one of the piped water supplies (location 8) had consistently lower chemical contamination indices than all other sources (Fig 1c and Table 2). The differences in chemical composition between locations, confirmed by pairwise comparisons between the sampling locations (Wilcoxon rank test, all *p*-values <0.05 with Bonferroni correction), suggest water mineralization and implicate contaminants such as vehicle exhaust gases [28,29] and poor waste handling systems as the key drivers of poor water quality in these locations [30].

**Table 2:** Summary of the chemical and bacterial contamination of the water sampling locations.

Source	Turbidity (NTU)	Chloride (mg/L)	Ammonia (mg/L)	Iron (mg/L)	Nitrite (mg/L as NaNO_2_)	Nitrate (mg/L as NaNO_2_)	MPN CFU/100ml	*S*. Paratyphi PCR positive (%)	*S*. Typhi PCR positive (%)
WHO*	0.5	250	1.5	0.3	3	50	0	-	-
1	0.5 (0.5, 2)	90.1 (82.2, 112)	0.2 (0.1, 0.4)	0.1 (0, 0.2)	0.04 (0.01, 0.15)	112.9 (98.3, 116.8)	33 (5, 3850)	25/40 (63)	30/40 (75)
2	0.5 (0.5, 1)	109.9 (89.1, 129.7)	0.2 (0.1, 0.33)	0.1 (0, 0.2)	0.02 (0.01, 0.06)	136.2 (119.6, 157.1)	1300 (207, 16750)	28/38 (74)	31/38 (82)
3	0.5 (0.5, 0.5)	83.2 (78.2, 88.1)	0.1 (0.1, 0.1)	0.1 (0, 0.2)	0.01 (0.01, 0.01)	142.7 (135.3, 158.5)	2 (1, 87)	38/51 (75)	40/51 (78)
4	58 (24.5, 115)	57.4 (55.4, 59.4)	10.5 (9.8, 11.2)	11.3 (8.7. 15.1)	0.06 (0.03, 0.13)	3.3 (1.25. 7.6)	1 (1, 2)	36/51 (71)	42/51 (82)
5	4 (2, 7.8)	87.1 (82.4, 94.1)	0.2 (0.1, 0.3)	0.2 (0.1, 0.3)	0.02 (0.01, 0.05)	117.7 (107.3, 127.2)	182 (6, 3025)	33/50 (66)	41/50 (82)
6	3 (2, 4)	169.8 (158.9, 174.6)	1.6 (0.9, 3.2)	0.12 (0.1, 0.5)	0.34 (0.19, 0.57)	191.1 (157.1, 204.5)	137 (7, 1700)	37/50 (74)	38/50 (76)
7	0.5 (0.5, 1)	83.2 (79.7, 90.1)	0.2 (0.1, 0.3)	0.01 (0, 0.2)	0.01 (0.01, 0.02)	93.3 (86.5, 106.0)	14 (2, 745)	39/51 (76)	43/51 (84)
8	2 (1, 2)	10 (9.9, 10.9)	0.1 (0, 0.2)	0.1 (0.1, 0.2)	0.01 (0.01, 0.01)	1.7 (1.6, 1.9)	1 (0, 1)	33/50 (66)	36/50 (72)
9	0.5 (0.5, 0.8)	66.3 (65.3, 68.3)	0.1 (0.1, 0.2)	0.01 (0, 0.2)	0.07 (0.05, 0.07)	94.1 (90.7, 96.3)	34 (7, 460)	15/21 (71)	13/21 (62)
10	0.5 (0.5, 0.6)	65.8 (64.1, 69.3)	0.1 (0, 0.2)	0.1 (0, 0.2)	0.02 (0.02, 0.05)	90.3 (82.4, 93)	31 (6, 2275)	19/30 (63)	19/30 (63)

*According to WHO guidelines [8], Data shown in medians and interquartile ranges unless described otherwise.

Of the different chemical pollutants observed in Kathmandu drinking water, the sustained contamination of water by nitrites and nitrates was the most alarming. Nitrites and nitrates can be introduced into the water through a range of processes including surface water infiltration, industrial pollution, agricultural fertilizer run-off and the leakage of sewerage systems [26,31]. Sustained exposure to nitrates can lead to a range of non-communicable diseases including methemoglobinemia, gastrointestinal cancer, bladder and ovarian cancers, and may expedite type II diabetes, thyroid hypertrophy and respiratory tract infections [32]. Nitrate excess is also a notable marker of fecal contamination, and we found nitrites and nitrates correlated positively with coliform concentration (see below) and weekly rainfall (*p<*0.001; Spearman’s rho). Consistently, the concentration of chloride, again a marker of sewage and manure contamination [33], also increased with the onset of seasonal rainfall (*p<*0.001; Spearman’s rho). More generally, several other chemical properties were also associated with weekly rainfall; positive correlations were observed for rainfall against turbidity, ammonia and hardness (Spearman’s rho, *p*-values <0.05 in six locations), and negative correlations were observed with rainfall against pH and alkalinity (Spearman’s rho, *p*-values <0.05 in four locations). Taken together, these results suggest that chemical pollution of drinking water in this setting is likely driven by a combination of rainfall runoff and localized contamination with human fecal waste.

### Water contamination with coliforms and *Salmonella* serovars Typhi and Paratyphi A

To determine the extent of potential fecal contamination in the water samples, we estimated the concentration of fecal indicator thermotolerant coliforms using the minimal probable number (MPN) method. The WHO guidelines state that no water directly intended for drinking, or in the distribution system should contain thermotolerant coliforms (in a 100ml sample) [8]. We found that majority of the cultured water samples were contaminated with thermotolerant coliforms, suggesting that all the sampled water sources are prone to fecal contamination (Table 2). The concentrations of cultured coliforms across the samples ranged from 0.1 to 2.5 x 10^8^ CFU/100 ml, these figures are again largely consistent with prior investigations of drinking water quality in this setting [8,26,31]. The water from the stone spouts (locations 1, 2, 3, 9 and 10 (Table 1) had higher coliform concentrations (median 94 CFU/100 ml; IQR: 4 to 1.6 x 10^4^) than that from the sunken wells (locations 4, 5, 6) (median 8 CFU/100 ml; IQR: 1 to 7.9 x 10^4^) and from the piped supplies (locations 7, 8) (Median 2 CFU/100 ml; IQR: 1 to 170) (*p<*0.001; Kruskal Wallis) (Fig 2a). The highest coliform concentrations across the sampled water sources were in the months of June, July and August and, similar to the chemical contamination, positively correlated with the period of increased weekly rainfall (Spearman’s rho *=* 0.27, *p<*0.001). The water sampled from source 2 (stone spout) consistently had the highest level of coliform contamination (median coliform concentration; 1.3 x 10^4^ CFU/100 ml; IQR: 200 to 1.68 x 10^5^) and had the highest single coliform count of 2.5 x 10^8^ CFU/100 ml in August.

The alarming levels of coliform and chemical contamination highlight that water quality is a major public health issue in Kathmandu. The detection of thermotolerant coliforms is not suitable for the identification of specific waterborne pathogens, but is used as a general measure of bacterial contamination [34]. To address this methodological limitation, we concurrently performed membrane filtration and microbiological enrichment for a range of pathogenic bacterial species classified as high risk by the WHO, including *Salmonella*, *Shigella*, *Vibrionaceae* and *E*. *coli* [8]. Overgrowth on plates diminished the ability to accurately quantify these organisms but we repeatedly cultured and identified a wide range of organisms with pathogenic potential to humans including *Vibrio cholerae* 01, *Shigella dysenteriae* type-1, *Pseudomonas* spp., and *Plesiomonas shigelloides* (S2 Table). We additionally isolated multiple *Salmonella* spp. in the water through supplementary enrichment, yet, after serotyping, none were identified as *S*. Typhi or *S*. Paratyphi A.

The lack of confirmative cultures for *S*. Typhi or *S*. Paratyphi A is not uncommon given previous efforts to culture these pathogens from water. *S*. Typhi has been cultured from environmental samples previously [35], but is notoriously difficult to isolate from water in endemic locations. It has been suggested that *S*. Typhi is often present and viable, but in a non-culturable state [36]. To address this limitation we extracted and purified total nucleic acid from each of the water samples after filtration and performed appropriately controlled quantitative real-time PCR on all samples for chromosomal targets specific for *S*. Typhi and *S*. Paratyphi A. We found that 333 (77%) and 303 (70%) of 432 DNA extractions from the eater samples were PCR amplification positive for *S*. Typhi and *S*. Paratyphi A, respectively (Fig 2b), with 266/432 (62%) samples being PCR amplification positive for both. To confirm that the PCR amplicons were *S*. Typhi and *S*. Paratyphi A, a random cross section of 96 (48 for each serovar) PCR amplicons were successfully cloned, sequenced and confirmed to originate from either *S*. Typhi or *S*. Paratyphi A. Further, through inference from standard curve, we identified a significant difference between the number of copies/reaction between *S*. Typhi (median 208 copies/reaction; IQR: 72 to 603) and *S*. Paratyphi A (median 11 copies/reaction; IQR: 4.6 to 28), corresponding with inferred significantly different medians of 4,200 and 2,200 copies/100 ml, respectively (*p*<0.001; Kruskal Wallis) (Fig 2b).

The presence of *S*. Typhi and *S*. Paratyphi A DNA in the water samples did not differ significantly across locations (non-parametric MANOVA: *Λ*_*pillai*_ = 0.020,*p* = 0.66 with 9,999 permutations). Therefore, we propose that processes of fecal contamination of water with *S*. Typhi and *S*. Paratyphi A operate non-locally and are likely to equally contaminate water sources in this area. This observation confirms previous work predicting that infection with a specific *S*. Typhi genotype is a random process [6]. This lack of geographical structure allowed us to pool data from all locations, gaining additional power to investigate the temporal trends of the average weekly PCR amplification profiles through a PCA. The first principal component (PC1) captured a gradient of *S*. Typhi and *S*. Paratyphi A DNA that exhibited a marked increase during weekly periods of rainfall in all water sources, and a subsequent decrease during the dry season (May–October) (Fig 2c). A simple seasonal model of PC1 (using a spline of the collection dates with five breakpoints) showed that this temporal trend was a significantly better fit than a model where PC1 was constant over time (ANOVA: *F* = 4.2184,*p* = 0.0034). Notably, the increased presence of *S*. Typhi and *S*. Paratyphi A in the water samples displayed a substantial temporal lag, with PC1 reaching a peak between one to two months after the end of the monsoon rains (Fig 2c). This delay may reflect the time taken for the organisms to reach the water outlet from the source of contamination or a concentration effect reflecting lower ground water levels in drier periods. We speculate that a leaking sewerage system and fluctuations in the pressure in the water supply pipes (negative pressure in the pipe results in an influx of sewage into the water pipe) are a likely source of this contamination.

### 16S rRNA gene surveying

While the trend of contamination by *S*. Typhi and *S*. Paratyphi A was similar across all sampled locations, these organisms likely represent a microscopic fraction of the diverse communities of microorganisms transiting through these water sources [37]. Assessing these complex microbial communities is relevant for investigating the extent of contaminating organisms present in these water sources and also aids tracing the likely sources of bacterial contamination. (i.e. soil and/or fecal waste). 16S rRNA gene surveying provides a suitably broad approach in conducting this type of investigation [38]. Therefore, the longitudinal structures of the bacterial communities in the two water sources with the greatest coliform concentrations (locations 2 and 5) were compared by 16S rRNA gene amplification and pyrosequencing (data available in S2 Dataset) [12]. The resulting 16S rRNA gene data showed that the bacterial communities in 93 tested water samples were composed of 11,212 OTUs, more than half of which were observed only once (S1 Fig). The bacterial diversity, as measured by Gini-Simpson’s index [39], was high in all analyzed samples (S2 Fig). We observed an increase in the average number of taxa found during the dry season (S3 Fig), suggesting that low rainfall induces a concentration effect and that exposure to a wider range of taxa likely increases during this period.

For a more comprehensive analysis, we investigated the bacterial diversity using a PCA of the 16S rRNA gene survey data transformed into OTU frequency profiles. This analysis showed that >80% of the variation amongst the analyzed samples could be summarized in seven dimensions, each representing a different assemblage of the detected OTUs (S4 Fig). Closer examination of the relative contributions of OTUs to each axis revealed that nearly identical principal components could be obtained through a combination of the 10 most common OTUs, which belonged to the genera *Acinetobacter* (OTUs 00001 and 0019), *Acidovorax* (OTU00011), *Comamonas* (OTU00025), *Flavobacterium* (OTU00042), *Bacillus* (OTUs 00503 and 00155), *Chryseobacterium* (OTU00208), *Staphylococcus* (OTU00436) and *Brevundimonas* (OTU00707) (S4 Fig).

The ten most commonly detected OTUs were comprised of bacterial genera that are typically found in the environment, and most are not recognized as enteric organisms. While many of these genera are known aquatic organisms, there is some overlap with genera that have recently been demonstrated to originate from sample preparation procedures [40]. To address this limitation, we specifically focused on the relative contributions of nine bacterial families that are common constituents of the gastrointestinal tract of mammals (*Bacteroidaceae*, *Clostridiaceae*, *Enterobacteriaceae*, *Erysipelotrichaceae*, *Lachnospiraceae*, *Lactobacillaceae*, *Prevotellaceae Ruminococcaceae* and *Veillonellaceae*) [41]. This sub-analysis (519 OTUs) showed that 80% of the variation could be summarized in only five dimensions (Fig 3a and 3b). Furthermore, almost identical principal components could be obtained through a combination of just six OTUs, which belonged to the *Enterobacteriaceae* (OTUs 00024, 00185 and 01479), *Bacteroidaceae* (OTU00979), *Clostridiaceae* (OTU00263), and *Prevotellaceae* (OTU 2149) (Fig 3c). We additionally found that the OTU composition was not random, and varied substantially between the sunken well and the stone spout. A DAPC analysis of the 16S rRNA gene data was used to identify combinations of the 519 gastrointestinal OTUs showing the greatest difference between the well (location 5) and the stone spout (location 2). The corresponding DAPC was able to recover the type of water source of the samples (based on their OTU composition) in 81% of cases (Fig 3c). The OTUs exhibiting the greatest variation (OTU00263; *Clostridium*, OTU00185; *Enterobacteriaceae*, OTU00979; *Bacteroides* and OTU00024; *Enterobacteriaceae*) were additionally four of the six principal fecal OTUs driving the temporal trends (Fig 3d). These results suggest the existence of local ecological factors, such as proximal sewage pipes, strongly affect the composition of bacterial communities in different types of water sources.

### The impact of contaminated water on the populous of Kathmandu

The current daily demand for water in Kathmandu is estimated to be 200,000 m^3^/d, but is unable to be met by municipal supplies, resulting in deficits of 70,000 to 115,000 m^3^/d in the wet and dry seasons, respectively [26]. This water deficit means that drinking water distribution lines operate at low pressure as compared to the overused sewage pipes that are often co-located. This pressure differential between sewage and drinking water lines, their relative proximity and poor state of repair accounts for the ingress of sewage into drinking water lines and an overall deterioration in their quality and safety. Furthermore, the cost associated with municipal water has led to an upsurge in the use of stone spouts in the middle and low-income residents of Kathmandu. The users of stone spouts now represent >20% of the water usage in Kathmandu [26]. Bacterial contamination has previously been shown to be higher in stone spouts than sunken wells than piped supplies in Kathmandu, with similar trends seen with nitrate contamination [26]. Our data confirm these associations and for the first time we show seasonal variations in fecal contamination, chemical pollutants and, vitally, exposure to both *S*. Typhi and *S*. Paratyphi A. We suggest that stone spouts are the most contaminated type of water source in this location as they are maximally exposed to potentially contaminating sources of human sewage. Further, the shallow aquifers supplying the stone spouts are likely more contaminated than deep aquifers, as they are, again, closer to the sources of potential contamination.

Our work shows that Kathmandu drinking water exhibits year round fecal contamination and is far from compliant with World Health Organization (WHO) standards [8] and it will be challenge to meet the United Nations sustainable development goal six (http://www.un.org/sustainabledevelopment/water-and-sanitation/#). It has been known for >100 years that the provision of filtered water has a dramatic effect on communicable diseases. In a classic paper by Sedgwick and McNutt they describe the observed decrease in mortality from typhoid fever and other infections when clean water is supplied to an urban population [42]. Taking these historic findings in account, we surmise that stone spouts and sunken wells represent a major public health risk to those in Kathmandu, not only for typhoid but also for other communicable and possibly non-communicable diseases. The chemical composition of drinking water indicates localized, site-specific pollution profiles, consistent with complex populations of enteric bacteria, which show both temporal and location specific profiles. For the first time we provide a molecular proxy for *S*. Typhi and *S*. Paratyphi A persistently transiting through an urban water source, where they appear to reach a peak concentration one to two months after the end of the monsoon. Whilst these results are very specific to this setting, many of the mechanisms facilitating such a large degree of contamination (water shortages, negative-pressure pipes, urban development, poor sanitation) are likely to be common in other typhoid endemic settings in Asia and beyond.

In conclusion, our work shows that municipal water in the capital city of Nepal exhibits evidence of substantial bacterial and chemical contamination. Further, we additionally show evidence of longitudinal contamination of both *S*. Typhi and *S*. Paratyphi A, demonstrating the impact of human fecal contamination and outlining that both these organisms are being continually transmitted through these water supply systems. Future research should focus on investigating the main routes of these bacterial and chemical pollutants into this water supply system. Further, the ability to culture and genotype organisms from the environment and human infection would close the circle on the role of the water delivery systems in urban settings for the transmission of typhoid fever. Nepal is amongst the poorest countries in Asia (GDP per capita of USD 703 in 2013 [43]) and substantial investment is required to improve the capacity and quality of the water supply in addition to the sewage handling systems in Kathmandu. As a rapid improvement of the water systems is unlikely to occur given the recent earthquake and ongoing political difficulties, we advocate the use of home water filters and sterilization systems alongside vaccination campaigns as the major public health interventions for typhoid fever prevention in this setting.

## Supporting Information

S1 Table The barcode and primer sequences (30 nucleotides for 454 adaptor, 12 nucleotides for unique recognition (tag) and 18 nucleotides to amplify the specific V3–V5 region) for 16S rRNA gene amplification and 454 sequencing. (DOCX)

S2 Table Pathogenic and non-pathogenic contaminating bacteria cultured from drinking water over the course of the investigation. (DOCX)

S1 Fig The distribution of the OTU frequencies in tested water samples. Bar plot showing that many OTUs are found in only one or two samples (left hand-side) while some rare OTUs are more common (found in 20+ samples, right hand-side).
(TIF)

S2 Fig Distribution of the Gini-Simpson index for bacterial diversity in tested water samples. Values on the x-axis correspond to the probability that two randomly chosen OTUs taken from a given water sample are different. Larger values represent greater diversity.
(TIF)

S3 Fig The relationship between bacteria diversity in water sample and rainfall. Scatter plot showing the non-linear relationship between bacteria richness (number of OTUs) and rainfall through time.
(TIF)

S4 Fig 16S rRNA gene surveying of all bacterial taxa found in stone spout and well water samples in Kathmandu, Nepal. a) Screeplot showing the eigenvalues of the PCA of 16S rRNA gene data, with retained axes in blue corresponding to 80% of the variation in 11,212 identified OTUs in the selected water samples. b) Diversity represented by the OTUs with large contributions to the retained PCA axes. PCA axes are represented on the x-axis, with a width proportional to the corresponding diversity (eigenvalue). The y-axis represents the amount of diversity retained by keeping only OTUs with a contribution exceeding a given threshold as indicated by the blue shades. The total surface of a given color is proportional to the fraction of the total diversity represented by this set of taxa. The red dashed line identifies the set of 10 retained taxa plotted in panel C, representing 80% of the total variation in the entire data. c) OTU composition of the water samples, showing the relative the ten most structuring fecal bacterial taxa identified in panel B (*Acinetobacter* (OTUs 00001 and 0019), *Acidovorax* (OTU00011), *Comamonas* (OTU00025), *Flavobacterium* (OTU00042), *Bacillus* (OTUs 00503 and 00155), *Chryseobacterium* (OTU00208), *Staphylococcus* (OTU00436) and *Brevundimonas* (OTU00707)). Their relative abundance (y-axis) is represented through time in location 2 and location 5, the two locations with the greatest estimated faecal contamination by MPN. Empty bars correspond to missing samples.
(TIF)

S1 Dataset Total bacterial and chemical data .
(XLSX)

S2 Dataset 16S rRNA data .
(XLSX)
