# Supplementary material for: The Ecological Dynamics of Fecal Contamination and Salmonella Typhi and Salmonella Paratyphi A in Municipal Kathmandu Drinking Water
Source: PLoS Negl Trop Dis. 2016 Jan 6;10(1):e0004346. doi: 10.1371/journal.pntd.0004346 (PMC4703202; doi:10.1371/journal.pntd.0004346)
Supplement: S1 Table — (DOCX) [file pntd.0004346.s001.docx]

**S1 Table**. The barcode and primer sequences (30 nucleotides for 454 adaptor, 12 nucleotides for unique recognition (tag) and 18 nucleotides to amplify the specific V3-V5 region) for 16S rRNA gene amplification and 454 sequencing

| **Sample ID** | **Barcode** | **Barcode + Reverse Primer (926R)** | **Forward Primer (338R)** |
| --- | --- | --- | --- |
| W05 - Wk1 | TAGTTGCGAGTC | TAGTTGCGAGTCCCGTCAATTCMTTTRAGT | ACTCCTACGGGAGGCAGCAG |
| W05 - Wk2 | TATACGCGCATT | TATACGCGCATTCCGTCAATTCMTTTRAGT | ACTCCTACGGGAGGCAGCAG |
| W05 - Wk3 | TATCAGGTGTGC | TATCAGGTGTGCCCGTCAATTCMTTTRAGT | ACTCCTACGGGAGGCAGCAG |
| W05 - Wk4 | TATCGCGCGATA | TATCGCGCGATACCGTCAATTCMTTTRAGT | ACTCCTACGGGAGGCAGCAG |
| W05 - Wk5 | TATCTCGAACTG | TATCTCGAACTGCCGTCAATTCMTTTRAGT | ACTCCTACGGGAGGCAGCAG |
| W05 - Wk6 | TATGCACCAGTG | TATGCACCAGTGCCGTCAATTCMTTTRAGT | ACTCCTACGGGAGGCAGCAG |
| W05 - Wk7 | TATGCGAGGTCG | TATGCGAGGTCGCCGTCAATTCMTTTRAGT | ACTCCTACGGGAGGCAGCAG |
| W05 - Wk8 | TATGGCACACAC | TATGGCACACACCCGTCAATTCMTTTRAGT | ACTCCTACGGGAGGCAGCAG |
| W05 - Wk9 | TATTCGTGTCAG | TATTCGTGTCAGCCGTCAATTCMTTTRAGT | ACTCCTACGGGAGGCAGCAG |
| W05 - Wk10 | TCAACAGCATCG | TCAACAGCATCGCCGTCAATTCMTTTRAGT | ACTCCTACGGGAGGCAGCAG |
| W05 - Wk11 | TCAATCTAGCGT | TCAATCTAGCGTCCGTCAATTCMTTTRAGT | ACTCCTACGGGAGGCAGCAG |
| W05 - Wk12 | TCACAGATCCGA | TCACAGATCCGACCGTCAATTCMTTTRAGT | ACTCCTACGGGAGGCAGCAG |
| W05 - Wk13 | TCACGATTAGCG | TCACGATTAGCGCCGTCAATTCMTTTRAGT | ACTCCTACGGGAGGCAGCAG |
| W05 - Wk14 | TCACTATGGTCA | TCACTATGGTCACCGTCAATTCMTTTRAGT | ACTCCTACGGGAGGCAGCAG |
| W05 - Wk15 | TCACTGGCAGTA | TCACTGGCAGTACCGTCAATTCMTTTRAGT | ACTCCTACGGGAGGCAGCAG |
| W05 - Wk16 | TCACTTCTCGCT | TCACTTCTCGCTCCGTCAATTCMTTTRAGT | ACTCCTACGGGAGGCAGCAG |
| W05 - Wk17 | TCAGACAGACCG | TCAGACAGACCGCCGTCAATTCMTTTRAGT | ACTCCTACGGGAGGCAGCAG |
| W05 - Wk18 | TCAGATCCGATG | TCAGATCCGATGCCGTCAATTCMTTTRAGT | ACTCCTACGGGAGGCAGCAG |
| W05 - Wk19 | TCAGCCATGACA | TCAGCCATGACACCGTCAATTCMTTTRAGT | ACTCCTACGGGAGGCAGCAG |
| W05 - Wk20 | TCAGCTCAACTA | TCAGCTCAACTACCGTCAATTCMTTTRAGT | ACTCCTACGGGAGGCAGCAG |
| W05 - Wk21 | TCAGGACTGTGT | TCAGGACTGTGTCCGTCAATTCMTTTRAGT | ACTCCTACGGGAGGCAGCAG |
| W05 - Wk22 | TCAGTACGAGGC | TCAGTACGAGGCCCGTCAATTCMTTTRAGT | ACTCCTACGGGAGGCAGCAG |
| W05 - Wk23 | TCAGTCGACGAG | TCAGTCGACGAGCCGTCAATTCMTTTRAGT | ACTCCTACGGGAGGCAGCAG |
| W05 - Wk25 | TCAGTGACGTAC | TCAGTGACGTACCCGTCAATTCMTTTRAGT | ACTCCTACGGGAGGCAGCAG |
| W05 - Wk26 | TCATCGCGATAT | TCATCGCGATATCCGTCAATTCMTTTRAGT | ACTCCTACGGGAGGCAGCAG |
| W05 - Wk27 | TCATCTGACTGA | TCATCTGACTGACCGTCAATTCMTTTRAGT | ACTCCTACGGGAGGCAGCAG |
| W05 - Wk28 | TCATGGTACACT | TCATGGTACACTCCGTCAATTCMTTTRAGT | ACTCCTACGGGAGGCAGCAG |
| W05 - Wk29 | TCCACGTCGTCT | TCCACGTCGTCTCCGTCAATTCMTTTRAGT | ACTCCTACGGGAGGCAGCAG |
| W05 - Wk30 | TCCAGTGCGAGA | TCCAGTGCGAGACCGTCAATTCMTTTRAGT | ACTCCTACGGGAGGCAGCAG |
| W05 - Wk31 | TCCGTCGTCTGT | TCCGTCGTCTGTCCGTCAATTCMTTTRAGT | ACTCCTACGGGAGGCAGCAG |
| W05 - Wk33 | TCCTAGCAGTGA | TCCTAGCAGTGACCGTCAATTCMTTTRAGT | ACTCCTACGGGAGGCAGCAG |
| W05 - Wk37 | TCCTCTGTCGAC | TCCTCTGTCGACCCGTCAATTCMTTTRAGT | ACTCCTACGGGAGGCAGCAG |
| W05 - Wk38 | TCCTGAGATACG | TCCTGAGATACGCCGTCAATTCMTTTRAGT | ACTCCTACGGGAGGCAGCAG |
| W05 - Wk39 | TCGAATCACAGC | TCGAATCACAGCCCGTCAATTCMTTTRAGT | ACTCCTACGGGAGGCAGCAG |
| W05 - Wk40 | TCGACTCCTCGT | TCGACTCCTCGTCCGTCAATTCMTTTRAGT | ACTCCTACGGGAGGCAGCAG |
| W05 - Wk41 | TCGAGACGCTTA | TCGAGACGCTTACCGTCAATTCMTTTRAGT | ACTCCTACGGGAGGCAGCAG |
| W05 - Wk43 | TCGAGCGAATCT | TCGAGCGAATCTCCGTCAATTCMTTTRAGT | ACTCCTACGGGAGGCAGCAG |
| W05 - Wk44 | TCGAGGACTGCA | TCGAGGACTGCACCGTCAATTCMTTTRAGT | ACTCCTACGGGAGGCAGCAG |
| W05 - Wk45 | TCGATACTTGTG | TCGATACTTGTGCCGTCAATTCMTTTRAGT | ACTCCTACGGGAGGCAGCAG |
| W05 - Wk46 | TCGATGAACTCG | TCGATGAACTCGCCGTCAATTCMTTTRAGT | ACTCCTACGGGAGGCAGCAG |
| W05 - Wk47 | TCGCATGAAGTC | TCGCATGAAGTCCCGTCAATTCMTTTRAGT | ACTCCTACGGGAGGCAGCAG |
| W05 - Wk48 | TCGCGTATTAGT | TCGCGTATTAGTCCGTCAATTCMTTTRAGT | ACTCCTACGGGAGGCAGCAG |
| W05 - Wk49 | TCGCTAGTGAGG | TCGCTAGTGAGGCCGTCAATTCMTTTRAGT | ACTCCTACGGGAGGCAGCAG |
| W05 - Wk50 | TCGGCTACAGAG | TCGGCTACAGAGCCGTCAATTCMTTTRAGT | ACTCCTACGGGAGGCAGCAG |
| W05 - Wk51 | TCGTACGTCATA | TCGTACGTCATACCGTCAATTCMTTTRAGT | ACTCCTACGGGAGGCAGCAG |
| W05 - Wk52 | TCGTCGATAATC | TCGTCGATAATCCCGTCAATTCMTTTRAGT | ACTCCTACGGGAGGCAGCAG |
| W05 - Wk53 | TCGTGATGTGAC | TCGTGATGTGACCCGTCAATTCMTTTRAGT | ACTCCTACGGGAGGCAGCAG |
| SS02 - Wk1 | TCGTGTCTATAG | TCGTGTCTATAGCCGTCAATTCMTTTRAGT | ACTCCTACGGGAGGCAGCAG |
| SS02 - Wk2 | TCGTTCACATGA | TCGTTCACATGACCGTCAATTCMTTTRAGT | ACTCCTACGGGAGGCAGCAG |
| SS02 - Wk3 | TCTACGGAGAGC | TCTACGGAGAGCCCGTCAATTCMTTTRAGT | ACTCCTACGGGAGGCAGCAG |
| SS02 - Wk5 | TCTACTCGTAAG | TCTACTCGTAAGCCGTCAATTCMTTTRAGT | ACTCCTACGGGAGGCAGCAG |
| SS02 - Wk6 | TCTAGCGTAGTG | TCTAGCGTAGTGCCGTCAATTCMTTTRAGT | ACTCCTACGGGAGGCAGCAG |
| SS02 - Wk7 | TCTAGTTAGTCG | TCTAGTTAGTCGCCGTCAATTCMTTTRAGT | ACTCCTACGGGAGGCAGCAG |
| SS02 - Wk8 | TGAACGCTAGCT | TGAACGCTAGCTCCGTCAATTCMTTTRAGT | ACTCCTACGGGAGGCAGCAG |
| SS02 - Wk9 | TCTCCGCATGTC | TCTCCGCATGTCCCGTCAATTCMTTTRAGT | ACTCCTACGGGAGGCAGCAG |
| SS02 - Wk10 | TCTCGTAATCAG | TCTCGTAATCAGCCGTCAATTCMTTTRAGT | ACTCCTACGGGAGGCAGCAG |
| SS02 - Wk11 | TCTCTAGAGCAT | TCTCTAGAGCATCCGTCAATTCMTTTRAGT | ACTCCTACGGGAGGCAGCAG |
| SS02 - Wk12 | TCTCTCCGTCGA | TCTCTCCGTCGACCGTCAATTCMTTTRAGT | ACTCCTACGGGAGGCAGCAG |
| SS02 - Wk13 | TCTGAGTCTGAG | TCTGAGTCTGAGCCGTCAATTCMTTTRAGT | ACTCCTACGGGAGGCAGCAG |
| SS02 - Wk14 | TCTGCGTACTAA | TCTGCGTACTAACCGTCAATTCMTTTRAGT | ACTCCTACGGGAGGCAGCAG |
| SS02 - Wk15 | TCTGCTAGATGT | TCTGCTAGATGTCCGTCAATTCMTTTRAGT | ACTCCTACGGGAGGCAGCAG |
| SS02 - Wk16 | TCTGTTGCTCTC | TCTGTTGCTCTCCCGTCAATTCMTTTRAGT | ACTCCTACGGGAGGCAGCAG |
| SS02 - Wk17 | TCTTAGACGACG | TCTTAGACGACGCCGTCAATTCMTTTRAGT | ACTCCTACGGGAGGCAGCAG |
| SS02 - Wk18 | TCTCACTAGGTA | TCTCACTAGGTACCGTCAATTCMTTTRAGT | ACTCCTACGGGAGGCAGCAG |
| SS02 - Wk19 | TGACATCAGCGG | TGACATCAGCGGCCGTCAATTCMTTTRAGT | ACTCCTACGGGAGGCAGCAG |
| SS02 - Wk20 | TGACCATATCGT | TGACCATATCGTCCGTCAATTCMTTTRAGT | ACTCCTACGGGAGGCAGCAG |
| SS02 - Wk21 | TGACGCGATGCA | TGACGCGATGCACCGTCAATTCMTTTRAGT | ACTCCTACGGGAGGCAGCAG |
| SS02 - Wk22 | TGACGGACATCT | TGACGGACATCTCCGTCAATTCMTTTRAGT | ACTCCTACGGGAGGCAGCAG |
| SS02 - Wk23 | TGAGACGTGCTT | TGAGACGTGCTTCCGTCAATTCMTTTRAGT | ACTCCTACGGGAGGCAGCAG |
| SS02 - Wk25 | TGAGAGAGCATA | TGAGAGAGCATACCGTCAATTCMTTTRAGT | ACTCCTACGGGAGGCAGCAG |
| SS02 - Wk26 | TGAGCACACACG | TGAGCACACACGCCGTCAATTCMTTTRAGT | ACTCCTACGGGAGGCAGCAG |
| SS02 - Wk27 | TGAGCGATTCTG | TGAGCGATTCTGCCGTCAATTCMTTTRAGT | ACTCCTACGGGAGGCAGCAG |
| SS02 - Wk28 | TGAGGATGATAG | TGAGGATGATAGCCGTCAATTCMTTTRAGT | ACTCCTACGGGAGGCAGCAG |
| SS02 - Wk29 | TGAGTCACTGGT | TGAGTCACTGGTCCGTCAATTCMTTTRAGT | ACTCCTACGGGAGGCAGCAG |
| SS02 - Wk30 | TGAGTTCGCTAT | TGAGTTCGCTATCCGTCAATTCMTTTRAGT | ACTCCTACGGGAGGCAGCAG |
| SS02 - Wk33 | TGATAGTGAGGA | TGATAGTGAGGACCGTCAATTCMTTTRAGT | ACTCCTACGGGAGGCAGCAG |
| SS02 - Wk37 | TGATCAGAAGAG | TGATCAGAAGAGCCGTCAATTCMTTTRAGT | ACTCCTACGGGAGGCAGCAG |
| SS02 - Wk38 | TGATGCTAACTC | TGATGCTAACTCCCGTCAATTCMTTTRAGT | ACTCCTACGGGAGGCAGCAG |
| SS02 - Wk39 | TGATGTGTGACC | TGATGTGTGACCCCGTCAATTCMTTTRAGT | ACTCCTACGGGAGGCAGCAG |
| SS02 - Wk40 | TGCAGAGCTCAG | TGCAGAGCTCAGCCGTCAATTCMTTTRAGT | ACTCCTACGGGAGGCAGCAG |
| SS01-Wk1 | TGCATTACGCAT | TGCATTACGCATCCGTCAATTCMTTTRAGT | ACTCCTACGGGAGGCAGCAG |
| SS01-Wk2 | TGCGCGAATACT | TGCGCGAATACTCCGTCAATTCMTTTRAGT | ACTCCTACGGGAGGCAGCAG |
| SS01-Wk3 | TGCGTATAGTGC | TGCGTATAGTGCCCGTCAATTCMTTTRAGT | ACTCCTACGGGAGGCAGCAG |
| SS01-Wk4 | TGCGTCAGTTAG | TGCGTCAGTTAGCCGTCAATTCMTTTRAGT | ACTCCTACGGGAGGCAGCAG |
| SS01-Wk5 | TGCGTGGTAGAC | TGCGTGGTAGACCCGTCAATTCMTTTRAGT | ACTCCTACGGGAGGCAGCAG |
| SS01-Wk6 | TGCTACCATGAG | TGCTACCATGAGCCGTCAATTCMTTTRAGT | ACTCCTACGGGAGGCAGCAG |
| SS01-Wk7 | TGCTAGTCATAC | TGCTAGTCATACCCGTCAATTCMTTTRAGT | ACTCCTACGGGAGGCAGCAG |
| SS01-Wk8 | TGCTATATCTGG | TGCTATATCTGGCCGTCAATTCMTTTRAGT | ACTCCTACGGGAGGCAGCAG |
| SS01-Wk9 | TGCTCAGTATGT | TGCTCAGTATGTCCGTCAATTCMTTTRAGT | ACTCCTACGGGAGGCAGCAG |
| SS01-Wk10 | TGCTCGTAGGAT | TGCTCGTAGGATCCGTCAATTCMTTTRAGT | ACTCCTACGGGAGGCAGCAG |
| SS01-Wk11 | TGCTCTAGTGGA | TGCTCTAGTGGACCGTCAATTCMTTTRAGT | ACTCCTACGGGAGGCAGCAG |
| SS01-Wk12 | TGCTGTGAGCTA | TGCTGTGAGCTACCGTCAATTCMTTTRAGT | ACTCCTACGGGAGGCAGCAG |
| SS01-Wk13 | TGGATATGCGCT | TGGATATGCGCTCCGTCAATTCMTTTRAGT | ACTCCTACGGGAGGCAGCAG |
| SS01-Wk14 | TGGCTCTACAGA | TGGCTCTACAGACCGTCAATTCMTTTRAGT | ACTCCTACGGGAGGCAGCAG |
| SS01-Wk15 | TGGTCATCACTA | TGGTCATCACTACCGTCAATTCMTTTRAGT | ACTCCTACGGGAGGCAGCAG |
| Negative Control | TGTACACGGCGA | TGTACACGGCGACCGTCAATTCMTTTRAGT | ACTCCTACGGGAGGCAGCAG |
