# Supplementary material for: The Ecological Dynamics of Fecal Contamination and Salmonella Typhi and Salmonella Paratyphi A in Municipal Kathmandu Drinking Water
Source: PLoS Negl Trop Dis. 2016 Jan 6;10(1):e0004346. doi: 10.1371/journal.pntd.0004346 (PMC4703202; doi:10.1371/journal.pntd.0004346)
Supplement: S2 Table — (DOCX) [file pntd.0004346.s002.docx]

**S2 Table.** Pathogenic and non-pathogenic contaminating bacteria cultured from drinking water over the course of the investigation

| **Bacterial genus** | **Species identified** | |
| --- | --- | --- |
| *Achromobacter* spp. |  | |
| *Acinetobacter* spp. ^B^ |  | |
| *Aeromonas* spp. ^B^ | *caviae*, *hydrophila*, *salmonicida*, *sobria* | |
| *Alcaligenes* spp. |  | |
| *Bordetella* spp. |  | |
| *Chromomonas* spp. | *violaceum* | |
| *Chryseomonas* spp. | *luteola* | |
| *Erwinia* spp. |  | |
| *Escherichia* spp. | *coli*, *coli* 1, *coli* 2, *fergusonii*, *hermanii*, *vulneris* | |
| *Flavobacterium* spp. ^B^ | *indologenes*, *oryzihabitans*, *meningosepticum* | |
| *Hafnia* spp. | *alvei* 1, *alvei* 2 | |
| *Klebsiella* spp. ^B^ | *oxytoca*, *planticola*, *pneumoniae* *ozaenae*, *pneumoniae* *pneumoniae*, *terrigena*, *ascorbata*, *cryocrescens* | |
| *Leclercia* spp. | *adecarboxylata* | |
| *Moraxella* spp. |  | |
| *Morganella* spp. | *morganii* | |
| *Pasteurella* spp. |  | |
| *Plesiomonas* spp. | *shigelloides* | |
| *Proteus* spp. | *mirabilis*, *vulgaris* | |
| *Providencia* spp. |  | |
| *Pseudomonas* spp. ^A,B^ | *aerugenosa* , *alcalifaciens*, *stuartii*, *rettgeri*, *fluorescens*, *cepacia*, *paucimobilis*, *pseudomallei*, *putida* | |
| *Rahnella* spp. | *aquatilis* | |
| *Salmonella* spp. ^A^ |  | |
| *Serratia* spp. ^B^ | *ficaria*, *fonticola*, *marcescens*, *plymuthica* | |
| *Shewanella* spp. | *putrefaciens* | |
| *Shigella* spp. ^A^ | *boydii*, *dysenteriae* 01, *flexneri*, *sonnei* | |
| *Sphingobacterium* spp. | *paucimobilis* | |
| *Tatumella* spp. | *ptyseos* | |
| *Vibrio* spp. ^A^ | *alginolyticus*, *cholerae* 01, *fluvialis*, *cholerae*, *metschnikovii*, *mimicus* | |
| *Weeksella* spp. | *virosa* | |
| *Xanthomonas* spp. | *maltophila* | |
| *Yersinia* spp. | *intermedia*, *rucker* | |
| ^A^ pathogens posing a substantial risk to human health | |  |
| ^B^ pathogens posing an opportunistic risk to human health | |  |
