# Supplementary figures and images for: The Ecological Dynamics of Fecal Contamination and Salmonella Typhi and Salmonella Paratyphi A in Municipal Kathmandu Drinking Water
Source: PLoS Negl Trop Dis. 2016 Jan 6;10(1):e0004346. doi: 10.1371/journal.pntd.0004346 (PMC4703202; doi:10.1371/journal.pntd.0004346)

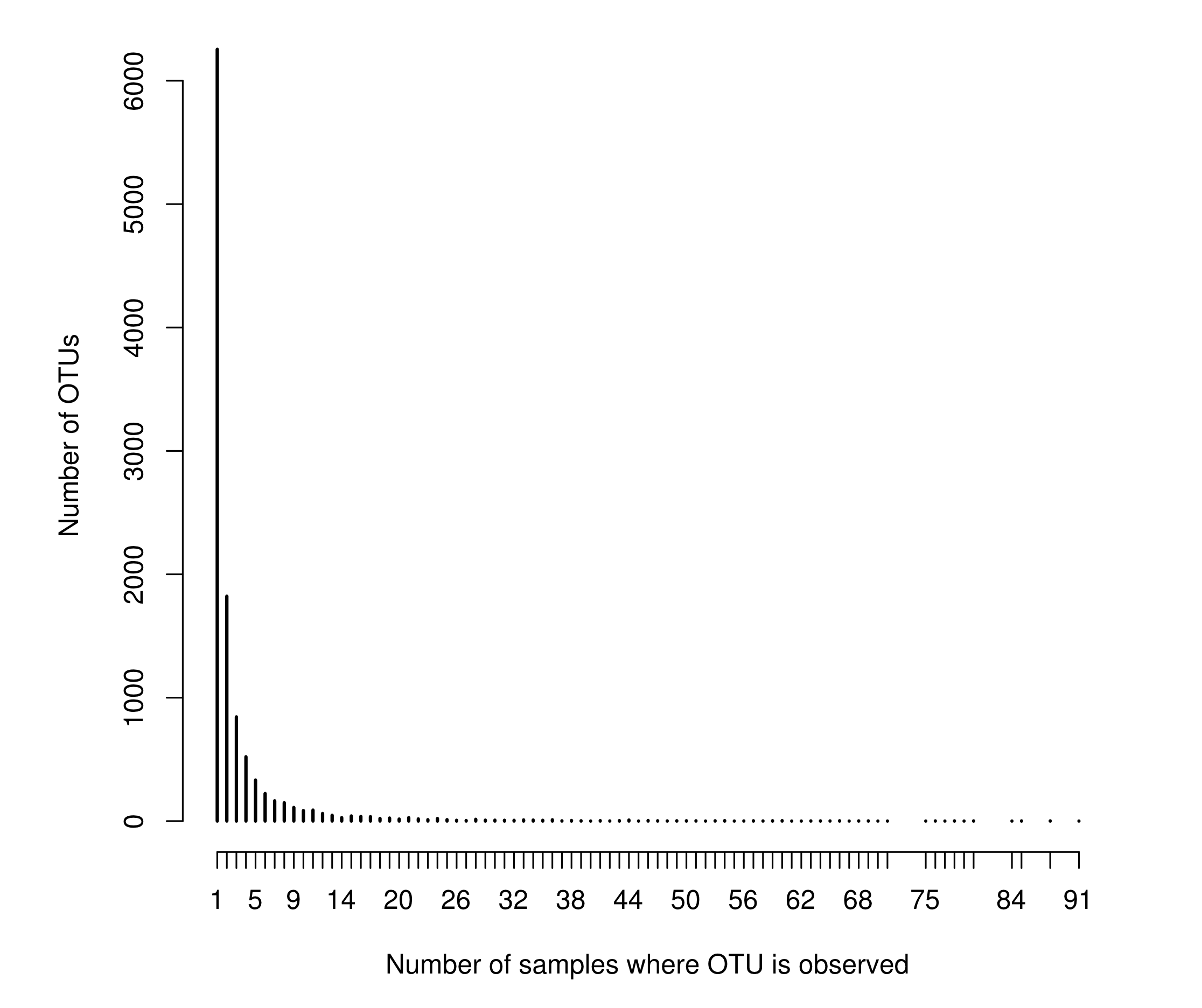

Supplement: S1 Fig — Bar plot showing that many OTUs are found in only one or two samples (left hand-side) while some rare OTUs are more common (found in 20+ samples, right hand-side). (TIF) [file pntd.0004346.s003.tif]

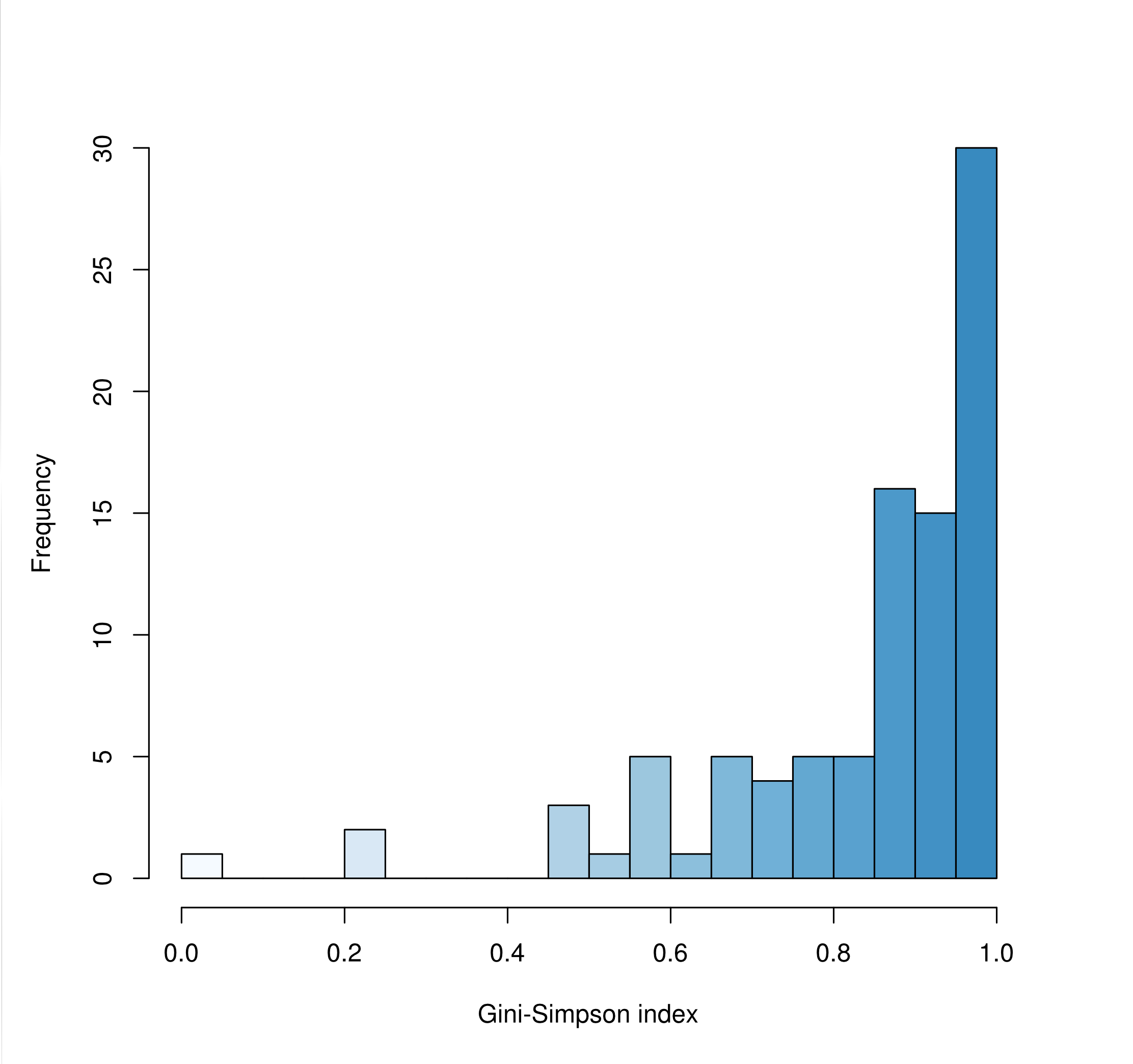

Supplement: S2 Fig — Values on the x-axis correspond to the probability that two randomly chosen OTUs taken from a given water sample are different. Larger values represent greater diversity. (TIF) [file pntd.0004346.s004.tif]

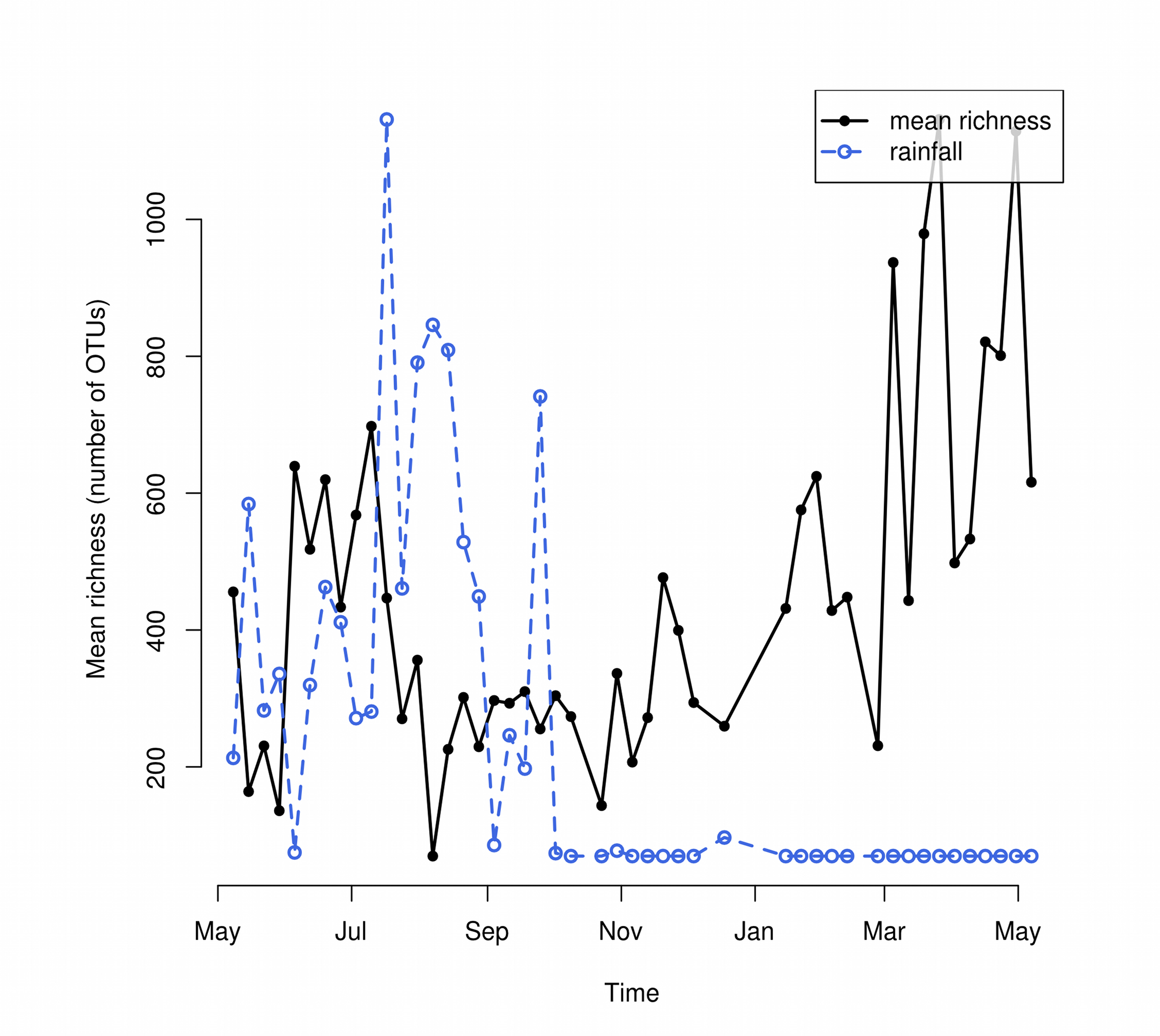

Supplement: S3 Fig — Scatter plot showing the non-linear relationship between bacteria richness (number of OTUs) and rainfall through time. (TIF) [file pntd.0004346.s005.tif]

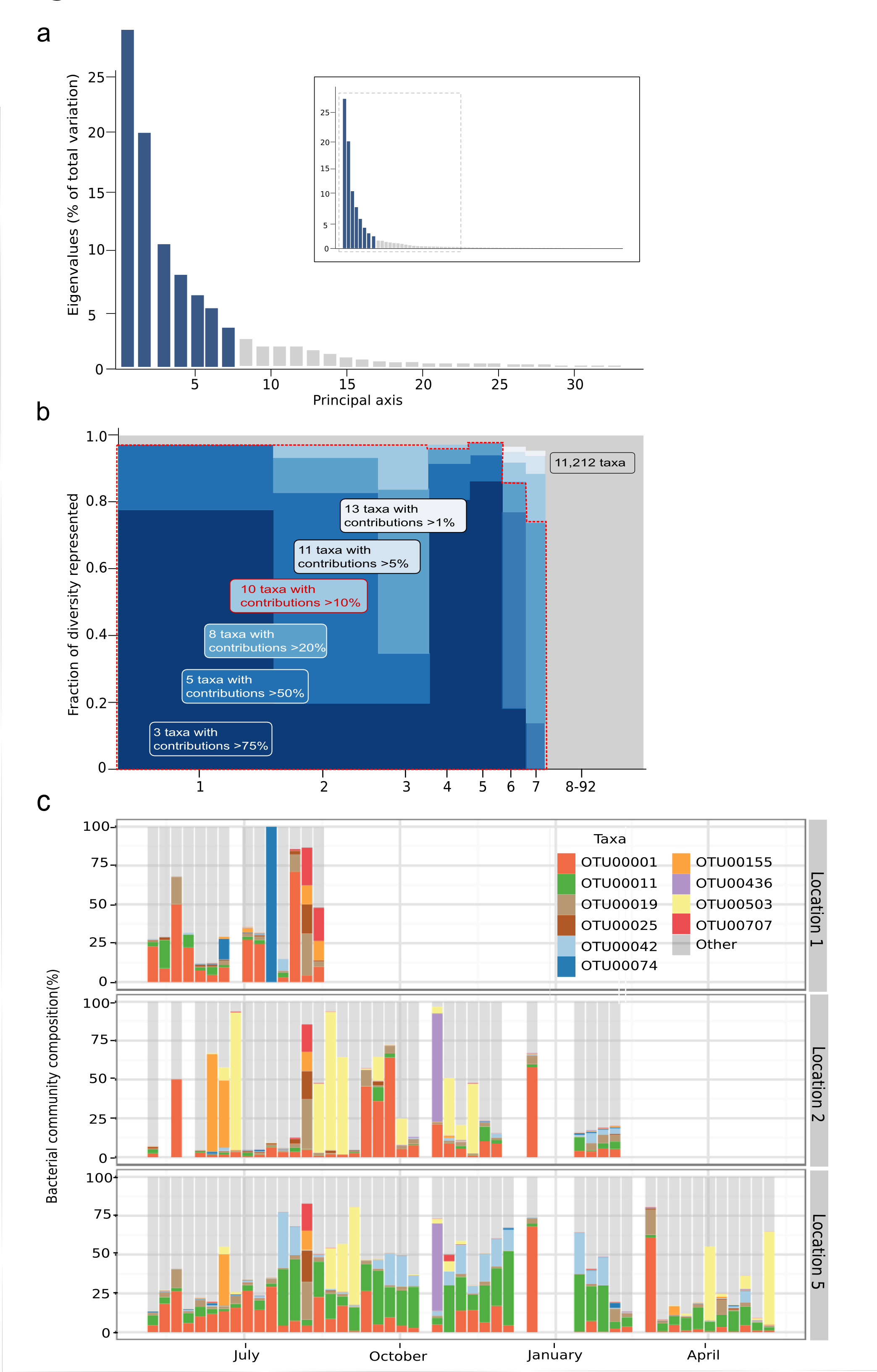

Supplement: S4 Fig — a) Screeplot showing the eigenvalues of the PCA of 16S rRNA gene data, with retained axes in blue corresponding to 80% of the variation in 11,212 identified OTUs in the selected water samples. b) Diversity represented by the OTUs with large contributions to the retained PCA axes. PCA axes are represented on the x-axis, with a width proportional to the corresponding diversity (eigenvalue). The y-axis represents the amount of diversity retained by keeping only OTUs with a contribution exceeding a given threshold as indicated by the blue shades. The total surface of a given color is proportional to the fraction of the total diversity represented by this set of taxa. The red dashed line identifies the set of 10 retained taxa plotted in panel C, representing 80% of the total variation in the entire data. c) OTU composition of the water samples, showing the relative the ten most structuring fecal bacterial taxa identified in panel B (Acinetobacter (OTUs 00001 and 0019), Acidovorax (OTU00011), Comamonas (OTU00025), Flavobacterium (OTU00042), Bacillus (OTUs 00503 and 00155), Chryseobacterium (OTU00208), Staphylococcus (OTU00436) and Brevundimonas (OTU00707)). Their relative abundance (y-axis) is represented through time in location 2 and location 5, the two locations with the greatest estimated faecal contamination by MPN. Empty bars correspond to missing samples. (TIF) [file pntd.0004346.s006.tif]
